# Supplementary material for: Applications of Blocker Nucleic Acids and Non-Metazoan PCR Improves the Discovery of the Eukaryotic Microbiome in Ticks
Source: Microorganisms. 2021 May 13;9(5):1051. doi: 10.3390/microorganisms9051051 (PMC8153336; doi:10.3390/microorganisms9051051)
Supplement: Supplementary file 1 [file microorganisms-09-01051-s001.zip › microorganisms-1200068-supplementary/Supplementary data/Supplementary Figures and Tables.pdf]

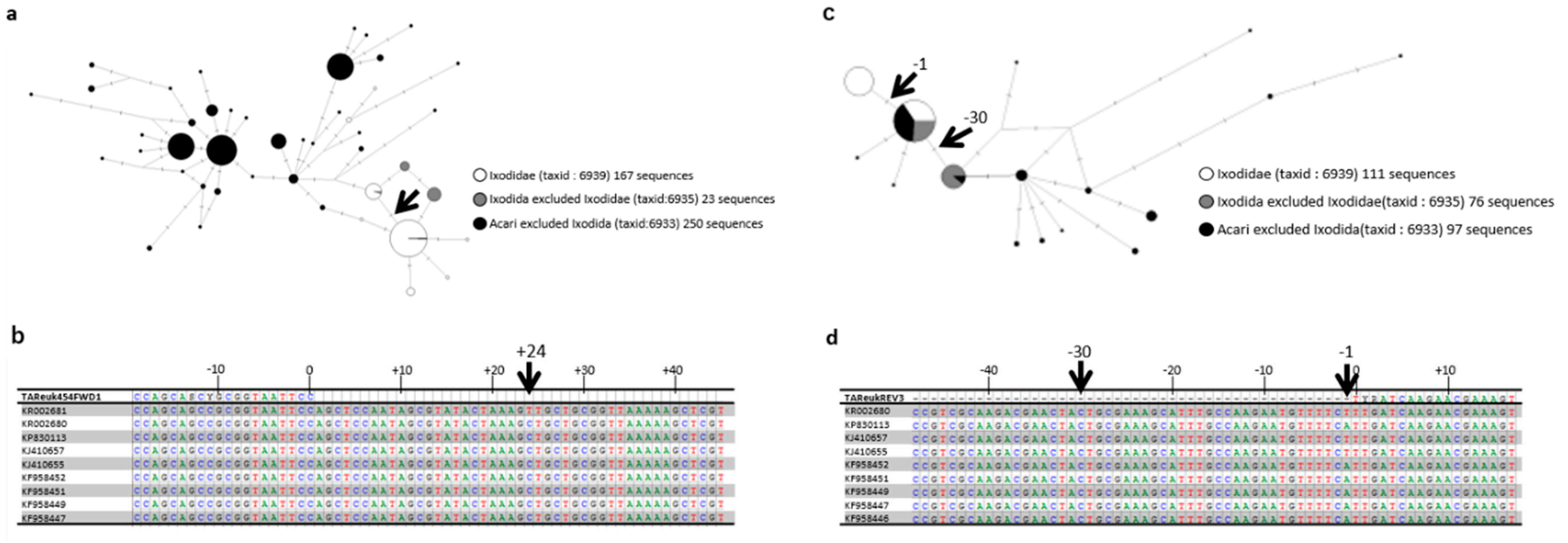

Figure S1. Median-joining network analysis for blocker designing. a) Median-joining network diagram of the TAReuk454FWD1 franking region at positions between 40 bp downstream and 1 bp upstream of the 3' end of the primer. The arrow indicates a single nucleotide mismatch at 24 bp downstream of the 3' end of the primer. b) Alignment of TAReuk454FWD1 and 18S rDNA sequences of the representative tick species. The left column in the table shows the primer ID or sequence accession number. The numbers at the top indicate the relative nucleotide position from the 3' end of the primer. c) Median-joining network diagram of the TAReukREV3 franking region at positions between 40 bp upstream and 1 bp downstream of the primer. The arrows indicate single nucleotide mismatch at 1 or 30 bp upstream of the primer. d) Alignment of TAReukREV3 and 18S rDNA sequences of the representative tick species. The left column in the table shows the primer ID or sequence accession number. The numbers at the top indicate the relative nucleotide positions from the 3' end of the primer.

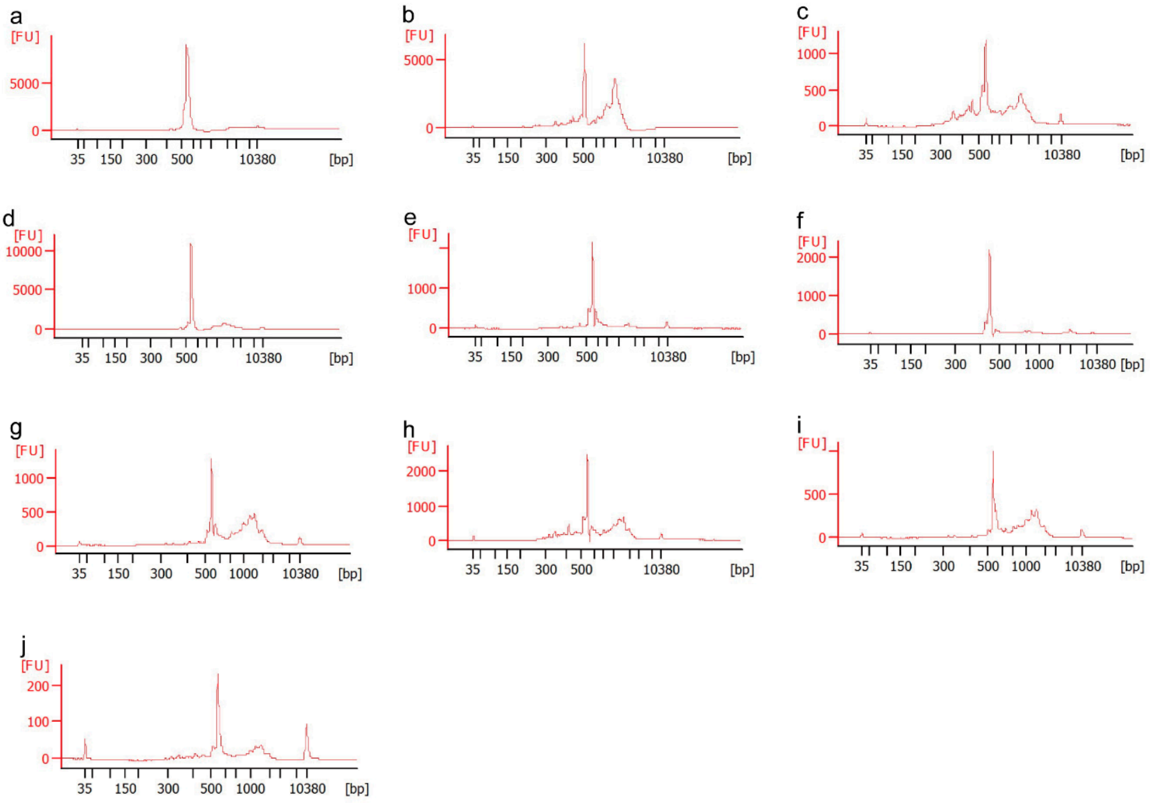

Figure S2. The fragment size distribution of the amplicon libraries using different PCR and purification methods. Amplicon libraries were prepared using *Ixodes persulcatus* DNA (tick ID: 933) (a-e) or *Amblyomma testudinarium* DNA (tick ID: 2874) (f-j). PCR and and purification methods were either control with AMPure (a and f), TickB_PNA with AMPure (b and g), TickB_PNA with SizeSelect (c and h), TickB_LNA with AMPure (d and i), or TickB_LNA with SizeSelect (e and j).

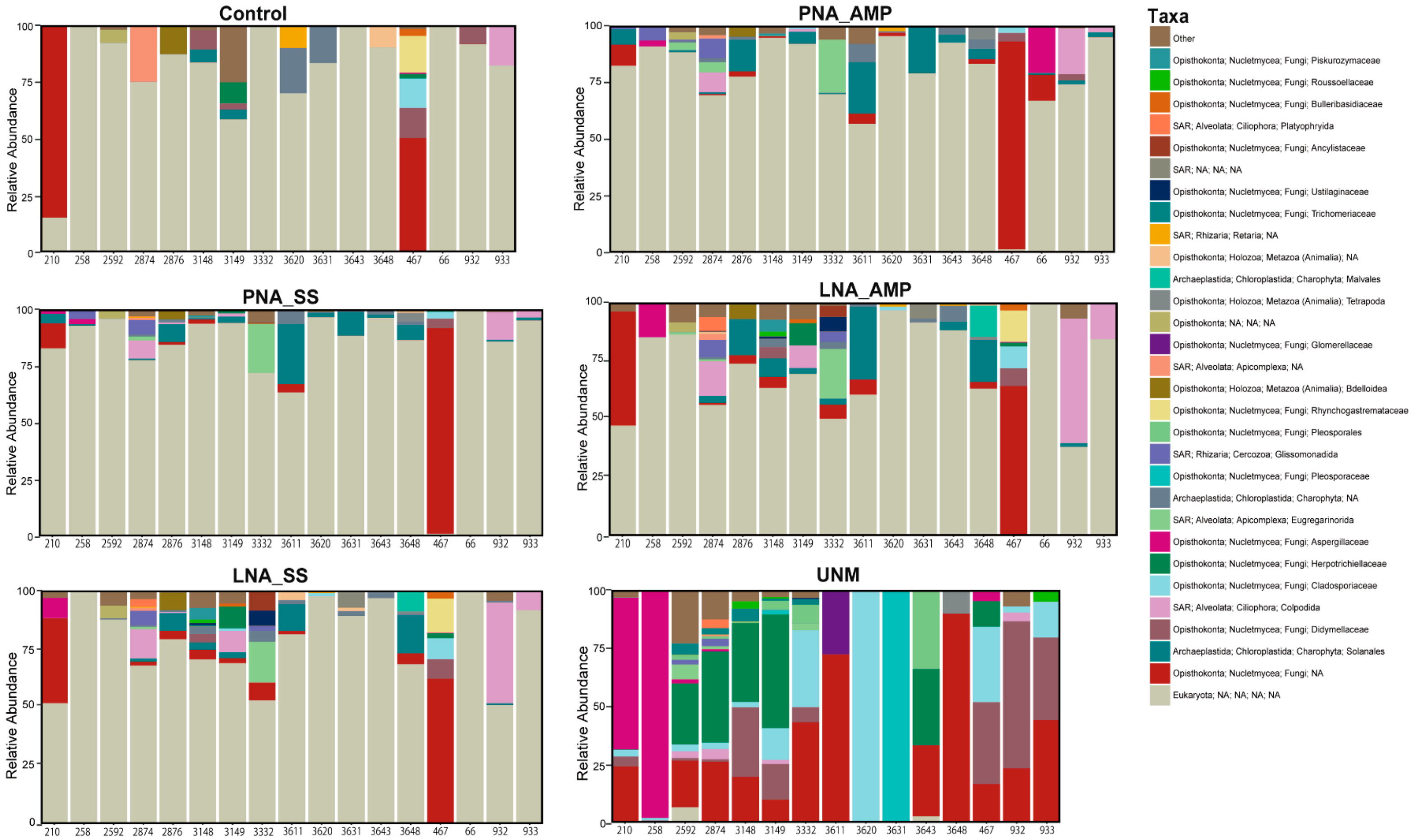

Figure S3. Relative abundance of eukaryotic taxa detected in 17 samples. PCR and purification methods for the amplicon analyses are provided on the top of bars. The PCRs were performed based on control, TickB_PNA (PNA), TickB_LNA (LNA), or UNonMet-PCR (UNM). The purification method was either with AMPure (AMP) or SizeSelect (SS). Each bar represents the eukaryotic taxa detected in one tick sample. The sample ID is provided on the bottom of each bar. Tick ID 3631 for control and tick IDs 66 and 2876 for UNM were excluded since no non-tick reads was obtained.

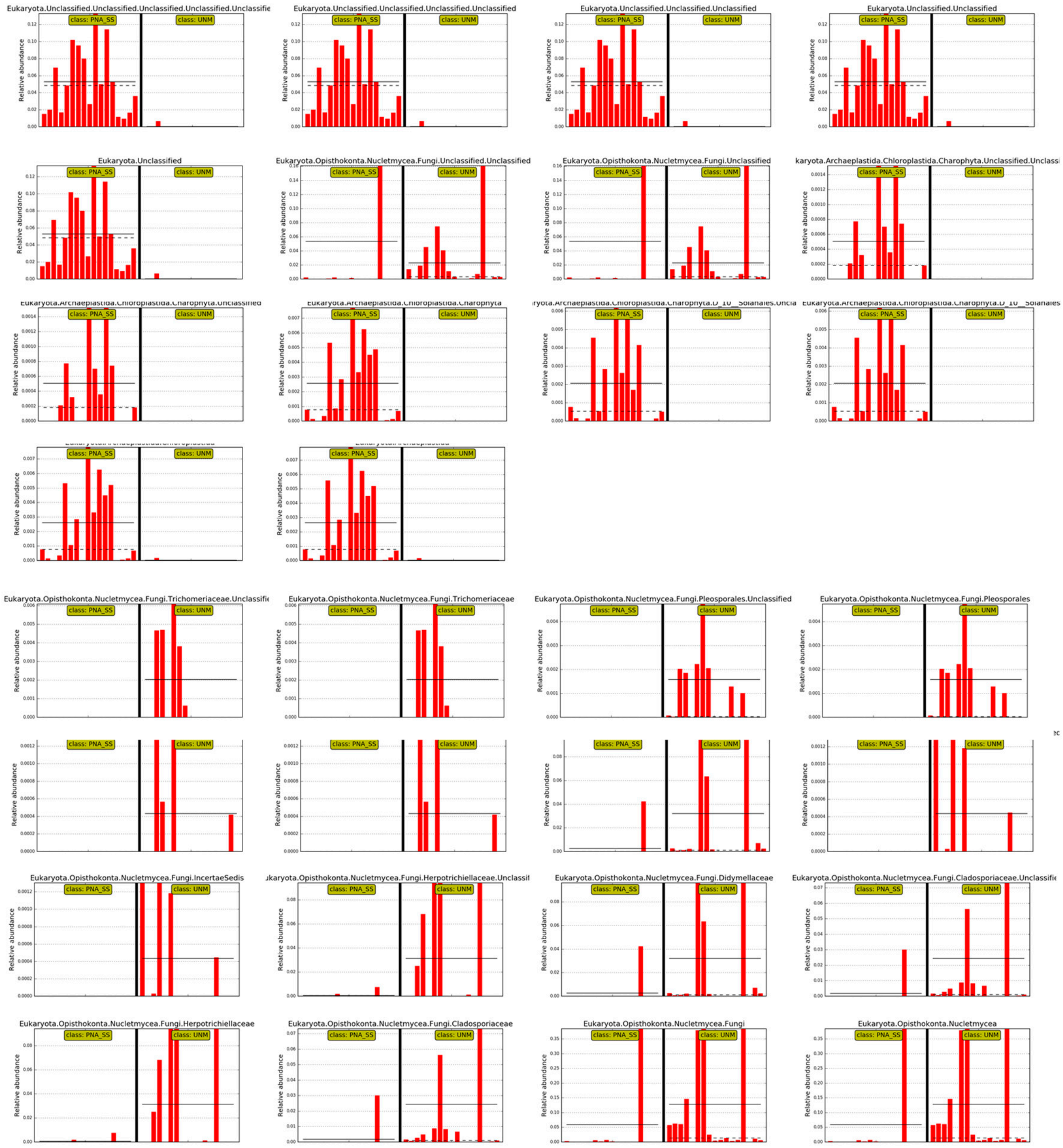

Figure S4. Significantly differential features between TickB_PNA and UNonMet-PCR based on LefSe analysis. Each graph indicates the differential features. The histogram indicates the relative abundance of each samples.

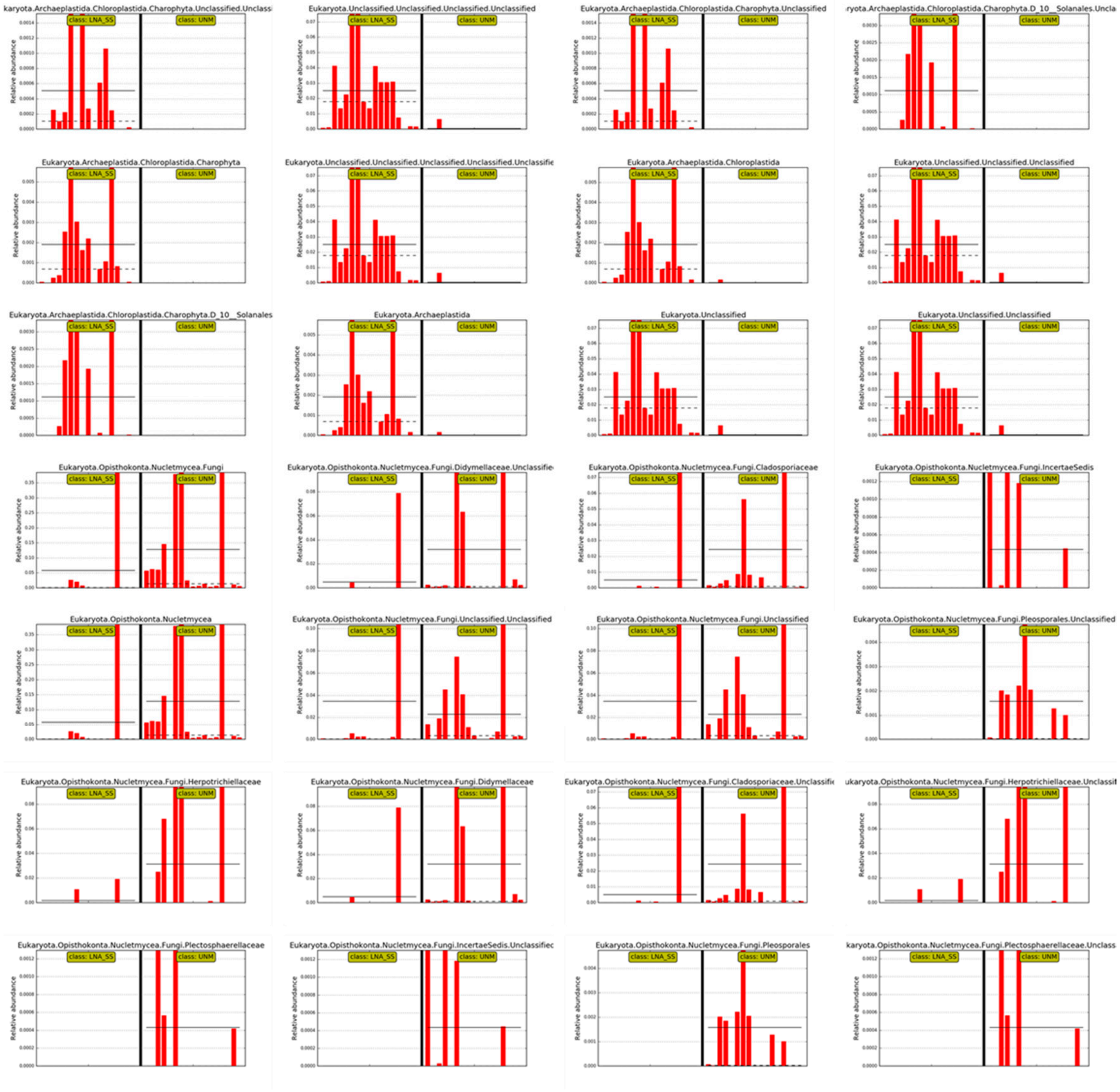

Figure S5. Significantly differential features between TickB_LNA and UNonMet-PCR based on LefSe analysis. Each graph indicates the differential features. The histogram indicates the relative abundance of each samples.

Table S2. The proportion of the non-tick reads and non-tick read enrichment value.

	Proportion of non-tick reads						Non-tick read enrichment values*				
**Tick ID**	**Control**	**PNA_AMP**	**PNA_SS**	**LNA_AMP**	**LNA_SS**	**UNM**	**PNA_AMP**	**PNA_SS**	**LNA_AMP**	**LNA_SS**	**UNM**
**66**	0.01%	0.75%	0.95%	0.01%	0.02%	0.00%	133.6	169.2	1.5	3.2	0.0
**210**	0.01%	1.57%	1.82%	0.09%	0.16%	5.74%	129.0	149.2	7.0	13.0	470.4
**258**	0.02%	1.52%	2.15%	0.09%	0.12%	6.23%	62.6	88.8	3.6	4.8	257.0
**467**	81.14%	99.14%	99.85%	94.39%	93.35%	99.56%	1.2	1.2	1.2	1.2	1.2
**932**	0.02%	1.01%	1.91%	0.25%	0.38%	1.11%	50.9	96.2	12.6	18.9	56.2
**933**	0.04%	1.56%	3.78%	0.13%	0.19%	0.63%	44.0	106.8	3.7	5.3	17.8
**2592**	0.21%	3.65%	7.22%	2.95%	4.72%	9.57%	17.4	34.5	14.1	22.6	45.8
**2874**	0.02%	1.49%	2.17%	1.32%	1.99%	17.44%	72.1	105.0	64.0	96.3	844.7
**2876**	0.06%	3.24%	5.75%	1.29%	2.83%	0.00%	58.0	102.7	23.0	50.6	0.0
**3148**	0.31%	5.41%	10.53%	8.64%	12.78%	37.90%	17.7	34.4	28.2	41.7	123.7
**3149**	0.49%	6.25%	10.14%	7.96%	11.71%	41.23%	12.8	20.8	16.3	24.0	84.6
**3332**	0.02%	8.59%	11.13%	2.36%	3.39%	2.52%	543.6	705.0	149.5	214.5	159.6
**3611****	0.00%	2.70%	4.18%	0.62%	1.67%	0.45%	-	-	-	-	-
**3620**	0.03%	9.96%	13.75%	2.39%	4.21%	0.67%	309.2	427.0	74.2	130.8	20.8
**3631**	0.06%	2.99%	5.63%	1.84%	3.43%	1.37%	46.7	87.9	28.7	53.5	21.4
**3643**	0.03%	7.70%	11.87%	1.53%	3.18%	0.38%	230.4	355.0	45.7	95.0	11.3
**3648**	0.03%	3.96%	6.09%	3.36%	4.53%	0.78%	125.8	193.7	106.9	144.0	24.7
**Median**	0.03%	3.24%	5.75%	1.53%	3.18%	1.37%	60.3	103.8	19.6	32.9	35.2
**Average**	4.85%	9.50%	11.70%	7.60%	8.74%	13.27%	115.9	167.3	36.3	57.5	133.7
**Max**	81.14%	99.14%	99.85%	94.39%	93.35%	99.56%	543.6	705.0	149.5	214.5	844.7
**Min**	0.00%	0.75%	0.95%	0.01%	0.02%	0.00%	1.2	1.2	1.2	1.2	0.0

Proportion of non-tick reads = non-tick reads / eukaryotic reads

*Non-tick read enrichment values = proportion of non-tick reads (each method) / proportion of non-tick reads (control)

**Non-tick read enrichment value was not calculated for tick ID 3611 because there was no non-tick reads in the control.

Table S3. Significance of Shannon Diversity.

Contrast	Estimate	Standard Error	t-value	p-value
**control - LNA_AMP**	-0.4728	0.163221	-2.8967	0.052233
**control - LNA_SS**	-0.75577	0.163221	-4.63037	0.000178
**control - PNA_AMP**	-0.96824	0.163221	-5.93205	8.54E-07
**control - PNA_SS**	-1.16467	0.163221	-7.13555	4.23E-09
**control - UNM**	-1.8932	0.165926	-11.4099	3.56E-10
**LNA_AMP - LNA_SS**	-0.28297	0.163221	-1.73366	0.513642
**LNA_AMP - PNA_AMP**	-0.49543	0.163221	-3.03534	0.036107
**LNA_AMP - PNA_SS**	-0.69187	0.163221	-4.23885	0.000765
**LNA_AMP - UNM**	-1.4204	0.165926	-8.56042	3.61E-10
**LNA_SS - PNA_AMP**	-0.21246	0.163221	-1.30168	0.783363
**LNA_SS - PNA_SS**	-0.4089	0.163221	-2.50518	0.133795
**LNA_SS - UNM**	-1.13743	0.165926	-6.85502	1.44E-08
**PNA_AMP - PNA_SS**	-0.19644	0.163221	-1.2035	0.834043
**PNA_AMP - UNM**	-0.92496	0.165926	-5.57456	3.94E-06
**PNA_SS - UNM**	-0.72853	0.165926	-4.39068	0.000439

Table S4. Significance of Faith's phylogenetic diversity.

Contrast	Estimate	Standard Error	t-value	p-value
**control - LNA_AMP**	-1.29551	0.207578	-6.2411	2.21E-07
**control - LNA_SS**	-1.67406	0.207578	-8.06474	4.07E-10
**control - PNA_AMP**	-2.00497	0.207578	-9.65887	3.56E-10
**control - PNA_SS**	-2.30394	0.207578	-11.0992	3.56E-10
**control - UNM**	0.348529	0.211019	1.651644	0.567053
**LNA_AMP - LNA_SS**	-0.37855	0.207578	-1.82364	0.456171
**LNA_AMP - PNA_AMP**	-0.70945	0.207578	-3.41776	0.011942
**LNA_AMP - PNA_SS**	-1.00843	0.207578	-4.85808	7.36E-05
**LNA_AMP - UNM**	1.644043	0.211019	7.790965	5.41E-10
**LNA_SS - PNA_AMP**	-0.3309	0.207578	-1.59412	0.604588
**LNA_SS - PNA_SS**	-0.62988	0.207578	-3.03444	0.036196
**LNA_SS - UNM**	2.02259	0.211019	9.584864	3.56E-10
**PNA_AMP - PNA_SS**	-0.29898	0.207578	-1.44031	0.70246
**PNA_AMP - UNM**	2.353495	0.211019	11.15299	3.56E-10
**PNA_SS - UNM**	2.652472	0.211019	12.56981	3.56E-10

Table S5. Significance of observed ASVs.

Contrast	Estimate	Standard Error	t-value	p-value
**control - LNA_AMP**	-0.35771	0.124878	-2.86449	0.056764
**control - LNA_SS**	-0.65776	0.124878	-5.26724	1.42E-05
**control - PNA_AMP**	-0.43484	0.124878	-3.48208	0.009797
**control - PNA_SS**	-0.69337	0.124878	-5.55236	4.33E-06
**control - UNM**	-0.58827	0.126945	-4.63406	0.000176
**LNA_AMP - LNA_SS**	-0.30005	0.124878	-2.40274	0.166612
**LNA_AMP - PNA_AMP**	-0.07712	0.124878	-0.61759	0.989474
**LNA_AMP - PNA_SS**	-0.33566	0.124878	-2.68786	0.087966
**LNA_AMP - UNM**	-0.23056	0.126945	-1.81622	0.460841
**LNA_SS - PNA_AMP**	0.222927	0.124878	1.785156	0.480541
**LNA_SS - PNA_SS**	-0.03561	0.124878	-0.28512	0.999738
**LNA_SS - UNM**	0.069489	0.126945	0.547394	0.993967
**PNA_AMP - PNA_SS**	-0.25853	0.124878	-2.07028	0.312376
**PNA_AMP - UNM**	-0.15344	0.126945	-1.20869	0.831528
**PNA_SS - UNM**	0.105095	0.126945	0.827871	0.961572

Table S7. The proportion of the unclassified eukaryotic reads to all eukaryotic reads.

Tick ID	Control	LNA-AMPure	LNA-SizeSelect	PNA-AMPure	PNA-SizeSelect	UNM
**66**	0.01%	0.01%	0.02%	0.50%	0.95%	0.00%
**210**	0.00%	0.04%	0.08%	1.30%	1.51%	0.00%
**258**	0.02%	0.07%	0.12%	1.38%	2.00%	0.00%
**467**	0.26%	0.34%	0.75%	1.47%	1.16%	0.00%
**932**	0.02%	0.10%	0.19%	0.75%	1.64%	0.00%
**933**	0.03%	0.11%	0.17%	1.48%	3.60%	0.00%
**2592**	0.19%	2.55%	4.13%	3.22%	6.92%	0.64%
**2874**	0.02%	0.74%	1.35%	1.03%	1.68%	0.03%
**2876**	0.05%	0.95%	2.24%	2.52%	4.85%	0.00%
**3148**	0.26%	5.48%	9.01%	5.12%	9.84%	0.00%
**3149**	0.29%	5.52%	8.07%	5.75%	9.53%	0.00%
**3332**	0.02%	1.19%	1.79%	6.00%	8.00%	0.00%
**3611**	0.00%	0.37%	1.35%	1.53%	2.65%	0.00%
**3620**	0.02%	2.31%	4.11%	9.50%	13.27%	0.00%
**3631**	0.05%	1.68%	3.05%	2.36%	4.98%	0.00%
**3643**	0.03%	1.35%	3.07%	7.13%	11.42%	0.01%
**3648**	0.03%	2.12%	3.10%	3.30%	5.27%	0.00%
**Average**	0.08%	1.47%	2.51%	3.20%	5.25%	0.04%
**Median**	0.03%	0.95%	1.79%	2.36%	4.85%	0.00%
**Min**	0.00%	0.01%	0.02%	0.50%	0.95%	0.00%
**Max**	0.29%	5.52%	9.01%	9.50%	13.27%	0.64%
